# Hospital Admission of Cancer Patients: Avoidable Practice or Necessary Care?

**DOI:** 10.1371/journal.pone.0120827

**Published:** 2015-03-26

**Authors:** Gianmauro Numico, Antonella Cristofano, Alessandro Mozzicafreddo, Olga Elisabetta Cursio, Pierfrancesco Franco, Giulia Courthod, Antonio Trogu, Alessandra Malossi, Mariella Cucchi, Zuzana Sirotovà, Maria Rosa Alvaro, Anna Stella, Fulvia Grasso, Silvia Spinazzé, Nicola Silvestris

**Affiliations:** 1 Medical Oncology and Hematology, Azienda USL della Valle d’Aosta, Viale Ginevra 3, 11100 Aosta, Italy; 2 University of Torino, Department of Oncology, Radiation Oncology Unit, Corso Bramante 88, 10126 Torino, Italy; 3 Medical Oncology Unit, National Cancer Research Center, Istituto Tumori "Giovanni Paolo II", Viale Orazio Flacco 65, 70124 Bari, Italy; University Campus Bio-Medico, ITALY

## Abstract

**Background:**

Cancer patients are frequently admitted to hospital due to acute conditions or refractory symptoms. This occurs through the emergency departments and requires medical oncologists to take an active role. The use of acute-care hospital increases in the last months of life.

**Patients and methods:**

We aimed to describe the admissions to a medical oncology inpatient service within a 16-month period with respect to patients and tumor characteristics, and the outcome of the hospital stay.

**Results:**

672 admissions of 454 patients were analysed. The majority of admissions were urgent (74.1%), and were due to uncontrolled symptoms (79.6%). Among the chief complaints, dyspnoea occurred in 15.7%, pain in 15.2%, and neurological symptoms in 14.5%. The majority of the hospitalizations resulted in discharge to home (60.6%); in 26.5% the patient died and in 11.0% was transferred to a hospice. Admissions due to symptoms correlated with a longer hospital stay and a higher incidence of in-hospital death.

**Conclusion:**

We suggest that hospital use is not necessarily a sign of inappropriately aggressive care: inpatient care is probably an unavoidable step in the cancer trajectory. Optimization of inpatient supportive procedures should be a specific task of modern medical oncology.

## Background

The landscape of the medical needs of cancer patients has undergone deep changes in the last decades that have not been followed by rapid changes in health care services. Cancer concerns an increasingly elderly population, often affected by multiple comorbidities [1]. Active treatments are offered for the majority of the natural history of the disease, and the exclusive palliative care phase has been shortened [2]. For some primary tumours available treatments are so aggressive that complex supportive care is needed: examples are chemo-radiotherapy treatments for head and neck, oesophageal and lung tumours, and chemotherapy for haematological diseases. On the other hand, while progressively improving in their organization and population coverage, home care services are not able to provide all the required assistance outside hospitals. Societal changes partially account for this greater need of care centralization. The consequence is that cancer patients frequently need hospital admission in acute care settings [3–6]. This progressive shift has two main consequences: 1) urgent admission is becoming a frequent modality of admission [4]. The more easy way to hospital seems to be the emergency department, which is open all day long, is able to provide comprehensive care and can rapidly refer the patient to the appropriate department. 2) The oncological wards, once intended to provide specialist care and complex active treatments, are frequently used for symptom management and for terminal care. While this trend has been attributed to inappropriate aggressiveness, especially in the last phase of life, or to an inadequate territorial coverage by palliative cares services [3], we hypothesized that the request of hospital admission and of acute care is independent from external factors and is somewhat unavoidable. As part of an internal quality improvement project we surveyed all the admissions in the onco-hematological ward of our General Hospital during a 16-month period. We were interested in understanding the characteristics of the patients admitted, the main reasons of admission, the interventions administered during their hospital stay, and the modalities of discharge.

## Patients and Methods

The Valle d’Aosta area, a special status Region in north-western Italy, has about 130,000 inhabitants (39.4/Km^2^). It is served by a single, 436-bed hospital that includes all surgical specialties, the emergency department, the intensive care unit and a radiotherapy unit with a tomotherapy equipment. The medical oncology department has an in-hospital acute care 12-bed ward. The inpatient oncology service admits patients with both solid and haematological tumours. As an internal policy the inpatient service is not used for chemotherapy administration unless the patient is at risk of medical complications (for example germ cell tumours or lymphoma with bulky disease undergoing their first treatment). The regional Palliative Care system includes a 7-bed hospice department together with home care and ambulatory services. Ambulatory patients are referred to the Palliative Care service as soon as there is not further indication to anti-cancer drugs and are fully cared for at home. Reasons for exclusion from home care are the lack of a stable caregiver and a concurrent severe medical acute condition. Intravenous treatments, oxygen and blood products can be delivered at home in the whole Region. Cancer patients referred to the emergency department are usually admitted to the oncological department unless they report conditions unrelated to cancer. A meeting with Palliative Care physicians is conducted twice-a-week in order to optimize in-hospital supportive care, select patients for hospice referral and arrange discharge for those able to be cared for at home.

All patients admitted to the General Valle d’Aosta Hospital inpatient oncology ward between August 1 2011 and December 31 2012, were included. Data were collected retrospectively and retrieved consecutively from the electronic medical records. Assessed patient characteristics included age, sex and site of primary cancer. The chief complaint at admission was recorded, together with the final diagnosis. Symptoms were clustered into 8 categories: breathlessness, pain, fever, intestinal obstruction, other digestive symptoms (nausea and vomiting, jaundice, diarrhea, dysphagia, etc.), neurological symptoms (mainly related to brain metastases or meningeal carcinomatosis), general symptoms (such as fatigue and cachexia) and cardiovascular symptoms (such as those related to deep vein thrombosis, pericardial effusion, heart failure). During hospitalization, information on the number and type of imaging studies, procedures, and antitumor interventions as well as length of stay and whether the admission was a repeated episode was collected. The modality of discharge was also recorded. The ethic committee of the “Azienda USL della Valle d'Aosta” approved this retrospective study and waived the requirement for informed consent. Patient informations were anonymized and de-identified prior to analysis. Data management has been conducted according to the principles expressed in the Declaration of Helsinki. All collected variables were described with simple statistics, such as medians and ranges, and the differences between groups were tested using the 2-sided Pearson Chi-square test, Fisher exact test, or Kruskal-Wallis test, as appropriate, with p<0.05 considered statistically significant. Data were analysed using the IBM SPSS Statistics for Windows, version 14.0 (IBM Corp., Armonk, NY).

## Results

Patient characteristics at admission are reported in Table 1. 672 admissions of 452 patients were performed in the examined period. While 297 patients were admitted once, 113 were admitted twice, 28 three times and 14 four or more times. 217 of the admissions (32,2%) occurred after at least one previous admission. Median time from one admission to the subsequent one was 6,8 weeks (range 1 to 80 weeks). In 19 (8,7%) and 58 (26,7%) cases the time from the subsequent admissions was 2 weeks or less and 4 weeks or less, respectively.

**Table 1:** Patient characteristics.

	Number	%
Number of admissions per patient		
One	299	66.1%
Two	115	25.4%
Three	28	6.1%
Four or more	11	2.4%
Sex		
Male	258	56.9%
Female	195	43.0%
Age		
Median age	69.2	
Range	26–92	
< 70	238	52.5%
≥ 70	215	47.4%
Site of primary		
Digestive tract	130	28.6%
Lung	114	25.1%
Hematologic	91	20.0%
Breast	33	7.2%
Urological	25	5.5%
Gynecological	20	4.4%
Other	30	6.6%

*the prevalent symptom at the time of admission was recorded.

258 of the cases were males (56.9%). 215 patients were aged 70 or older (47.4%). Three cancer types accounted for the vast majority of admitted patients (73.7%): digestive tract (28.6%), lung (25.1%) and haematological (20.0%).

Characteristics of admissions are detailed in Table 2. Patients were admitted with the purpose of performing complex diagnostic or therapeutic procedures in 19.8% of the cases. In all other cases (80.2%) a prevalent symptom was the cause of admission. Among the admissions due to symptoms, the chief complaint was breathlessness in 15.7%, pain in 15.2% and neurological symptoms in 14.5%. The most common modality of referral to the oncological ward was an urgent admission (74.1%). A programmed admission or a transfer from other units was far less common (12.4% and 13.5% respectively). Of the 498 urgent admissions, 329 (66.1%) were referred through the emergency department, while 169 (33.9%) were referred directly by the oncological or palliative care services.

**Table 2:** Features of admissions.

	Number	%
Reason for admission		
Cancer diagnosis or treatment	133	19.8%
Symptom*	539	80.2%
Dyspnea	85	15.7%
Pain	82	15.2%
Neurological	78	14.5%
Fever	72	13.3%
Digestive tract (vomiting, jaundice…)	72	13.3%
Intestinal obstruction	67	12.4%
General (asthenia, malaise…)	66	12.2%
Cardiovascular	17	3.1%
Modality of admission		
Programmed	83	12.4%
Transferred from other units	91	13.5%
Urgent	498	74.1%
Diagnosis		
Tumor-related condition	499	74.3%
Treatment-related toxicity	51	7.6%
Infection	95	14.1%
Vascular event	27	4.0%
Treatment		
Medical supportive measures only	382	56.8%
Chemotherapy	51	7.6%
Invasive procedures	224	33.3%
Radiotherapy	15	2.2%
Length of stay		
Mean (days)	12.1	
Median (days)	9.0	
Range	0–66	
≤ 7 days	282	42.0
> 7 days	390	58.0
Discharge		
At home	407	60.6%
In-hospital death	178	26.5%
Hospice or long-term medical care	87	12.9%

*the prevalent symptom at the time of admission was recorded.

In 74.3% of the admissions a condition that was directly related to tumor involvement was diagnosed. A toxic event due to chemotherapy or chemo-radiotherapy was the cause of admission in 7.6%. Cancer-related Infections accounted for the 14,1% of the admissions while cardiovascular events were 4,0%.

During their hospital stay the majority of the patients received only medical supportive measures (such as antibiotics for infection or opiates for pain); 33.3% had a diagnostic or therapeutic invasive procedure performed (such as thoracentesis for pleural effusion or biliary drainage for obstruction). Only 9.8% of the patients received chemotherapy or radiotherapy. Median duration of hospital stay was 9.0 days, with 58.0% of the admissions having a duration of more than 7 days. One-hundred and seventy-eight admissions (26.5%) ended with in-hospital death and another 12.9% was transferred to hospice.

When compared with admissions due to other reasons (Table 3), admissions due to symptoms were more frequently urgent, were longer, and resulted more frequently in the patient’s death. When primary sites were analysed, digestive tract and lung tumors were more frequent in the symptomatic population, while haematological cancers were prevalent in the admissions of non-symptomatic patients.

**Table 3:** Analysis of admissions divided per reason of admission: symptoms or other reasons.

	Symptoms (539)	Other reasons (133)	P
Sex			0.483
Male	302 (56.0%)	79 (59.4%)	
Female	237 (44.0%)	54 (40.6%)	
Age			0.244
<70	294 (54.5%)	80 (60.2%)	
>70	245 (45.5%)	53 (39.8%)	
Site of primary			0.0001
Digestive tract	157 (29.1%	34 (25.6%)	
Lung	136 (25.2%)	26 (19.5%)	
Hematologic	94 (17.4%)	50 (37.6%)	
Breast	44 (8.2%)	5 (3.8%)	
Urological	32 (5.9%)	5 (3.8%)	
Gynecological	29 (5.4%)	5 (3.8%)	
Other	47 (8.7%)	8 (6.0%)	
Length of stay			0.001
<7 days	210 (39.0%)	72 (54.1%)	
>7 days	329 (61.0%)	61 (45.9%)	
Modality of admission			0.0001
Programmed	33 (6.1%)	50 (37.6%)	
Transferred	40 (7.4%)	51 (38.3%)	
Urgent	466 (86.5%)	32 (24.1%)	
Diagnosis			0.0001
Tumor-related condition	378 (70.1%)	121 (91.0%)	
Non tumor-related condition	114 (21.2%)	8 (6.0%)	
Treatment-related toxicity	47 (8.7%)	4 (3.0%)	
Discharge			0.0001
At home	306 (56.8%)	101 (75.9%)	
In-hospital death	162 (30.1%)	16 (12.0%)	
Hospice or long-term medical care	71 (13.2%)	16 (12.0%)	

Among the prevalent symptoms recorded at admission, pain, bowel obstruction and neurological symptoms caused the longest hospital stays (median number of days: 12.5, 11.0 and 11.0 respectively); breathlessness and general symptoms were more frequently associated with hospital death (42.4 and 53.0%, respectively).

## Discussion

The reported data clearly suggest that the medical oncology in-hospital ward is largely used for symptom control in advanced cancer patients. This implies that admissions are frequently unscheduled, are repeated, are quite long and result in patients’ death or palliative care referral in more than one third of cases. This scenario calls for a reflection on the skills required by medical oncologists and about the provision of a comprehensive care organization for cancer patients.

The first issue to discuss is whether these data are reliable and how our findings compare with those of other recent reported series. Several reports contribute to the growing awareness that cancer care often involves dealing with acute conditions and symptom control. In a large series of unscheduled ambulatory consultations to another Italian oncological service, pain (27.7%), fatigue (17.6%), dyspnea (13.8%) and fever (11.5%) were the more frequent reasons for the visit request [7]. Authors from the University of Wisconsin [8] reported on 149 unplanned oncology admissions in 2010: in 66% of the cases patients were admitted due to symptoms and only in 3% for chemotherapy administration. Pain was the most common chief compliant at admission (28%). Time of hospital stay increased as the number of admissions finalized to chemotherapy administration decreased in a ten-year period. The rate of referral to hospice was 12% (compared to 18% in our series). These data indicate that the oncology in-patient services are increasingly used for managing acute cancer-related conditions.

A second important issue is whether these admissions should be minimized through referral of patients to other services or physicians. Could the improvement of cancer care reduce the need for acute-hospital referral? Several lines of evidence suggest that the expansion of home care and hospice use does not reduce ED and ICU referrals nor hospitalization due to acute conditions [9]. Cancer patients have unpredictable and complex medical needs that in some instances can be managed only in the context of in-patient services. Researchers from the Dana Farber Cancer Institute assessed avoidable hospitalization in 201 admissions of patients with gastro-intestinal malignancies and, through a retrospective review of patients’ health records, found that only 19% of the admissions could have been avoided [10]. Studies assessing emergency department referral of oncological patients support the hypothesis that the majority are unavoidable and result in hospital admission [11–12]. A recent comment about the increasing rate of hospital readmission among Medicare patients [13] suggest “hospital-dependence” as a common status of patients whose medical problems “cannot be managed outside the hospital”. Although there may be some place for improvement, our data also support the hypothesis that acute events cannot be managed outside hospital. Every effort to improve territorial services is limited by the occurrence of acute events and the overlapping of multiple conditions (symptoms, organ failure, psychological discomfort, social frailties) that cannot be managed without hospital care [14].

At the present time, the theory that better home care causes reduced hospital use is not evidence-based and proponents have not been able to demonstrate this relationship [11, 15].

Instead we propose that the treatment of acute events and refractory symptoms should be considered a specific task of oncology services: improving quality of the cares provided, shortening the time to symptom control and appropriately using both medical and invasive treatments are probably the aim of a modern oncology ward.

Admission of cancer patients through the emergency department may be viewed as uncomfortable due to the possibly long waiting times and clinical assessment provided by physicians who do not usually take care of the patient. Moreover, partial patient knowledge could induce inappropriately aggressive interventions. Anticipating the critical worsening of symptoms by booking admission in advance and favouring direct admissions from home and ambulatory services are probably two possible ways of improving patient care. In our series one out of three admissions was driven without access to the ED, while in other series this practice seems rare [15]. Providing an acute oncological consulting service could further improve this process [7, 16]. However, in the majority of the cases, the need for timely, multiple laboratory and radiological examinations and for a comprehensive, coordinated assessment make the emergency department the only adequate response to the patients’ needs in complex conditions.

The final issue is whether the high rate of death in an acute care setting could be reduced. In our series one third of admissions resulted in in-hospital death or hospice referral. Could these patients have been more appropriately assisted at home or referred directly to a hospice? We have already shown that prognostication in the context of an acute event is not straightforward [17]. The frequency of hospitalization near the end of life has been shown to be increasing, and has been included among the indicators of aggressive care, thus suggesting that there is an inverse relationship between the rate of hospital admission and the quality of palliative care organization [18]. However, studies assessing the influence of home palliative cares, while showing significant differences in terms of place of death [19–20], did not find a reduction in hospital admissions or emergency department visits [9, 21–22]. The majority of patients with cancer still die in hospitals in several European countries [23] and hospitalization in the last period of life is extremely common. An analysis of hospital use conducted in Ontario on more than 200.000 cancer patients who died between 1986 and 1998 found that less than 10% were not admitted to hospital in the last 6 months of life and that the hospitalization rate dramatically increased as death approached [24]. Similar data have also been found in European healthcare systems [25–27]. This is in line with our opinion that complex symptoms or acute events cannot be fully cared for by home services. Moreover home care is strictly dependent from the familiar and social environment: as the potential caregivers are progressively less able to take care of their relatives (mainly due to socio-economic factors) the hospital becomes the only possible solution for terminal care. For these reasons, focusing health care policy strictly on organizing end-of-life care at home may not decrease hospital referral of cancer patients [28] and may not decrease costs of advanced cancer care [29].

We suggest that in-hospital care of symptoms and acute events should be improved in order to provide prompt palliation and clinical stabilization. Afterwards, care should be kept on in low-intensity structures. Flexibility of the system, with easy transitions from one care setting to another, is probably the goal to be pursued, instead of closing the doors of hospitals to advanced cancer patients or considering the referrals to the emergency departments as inappropriate.

Duration of in-hospital stay should be optimized through improved and faster symptom control. In our experience longer stays were those in which admission was due to pain, bowel obstruction and neurological symptoms. Although variable reasons could be implicated, we support the hypothesis that adoption of evidence-based procedures may reduce the time to symptom control.

Some limitations of our study must be taken into account. As this is an evaluation of a small Italian region, with a single oncological and palliative care unit, the data may not be fully generalizable. For example the organization of home services and social support, and the formal and informal rules applied in the hospital context could have influenced the results of our analysis, although we have shown that comparable issues are reported in other contexts. Finally, the retrospective nature of the study carries some inherent biases such as the incomplete recording of some patient-related factors and the possible exclusion of a few cancer patients admitted to other departments during the examined period.

In conclusion, the data shown in this report support the crucial role of oncologists in the context of inpatient care. Competences and skills should be enhanced accordingly and should be considered part of the specific place of oncology in cancer care [30]. Moreover we suggest that hospitalization of cancer patients should be considered a necessary step in the trajectory of the disease [31]; rather than considering hospitalization as avoidable or even inappropriate, we should increase our efforts to assure rapid symptom control and prompt stabilization of acute conditions.
